# MiRNA-497 regulates cell growth and invasion by targeting cyclin E1 in breast cancer

**DOI:** 10.1186/1475-2867-13-95

**Published:** 2013-10-10

**Authors:** Qifeng Luo, Xiaoyu Li, Yan Gao, Yin Long, Lei Chen, Yixiang Huang, Lin Fang

**Affiliations:** 1 Department of General Surgery, Shanghai Tenth People’s Hospital, Tongji University School of Medicine, Shanghai 200072, P.R. China; 2 Department of Clinical Laboratory Medicine, Shanghai Tenth People’s Hospital, Tongji University School of Medicine, Shanghai 200072, P.R. China

**Keywords:** MiR-497, Breast cancer, Cyclin E1

## Abstract

**Background:**

MicroRNAs are a class of endogenous single strand non-coding RNAs that are involved in many important physiological and pathological processes. The purpose of this study was to examine the expression levels of miR-497 in human breast cancer and its function in MDA-MB-231 breast cancer cells.

**Methods:**

Quantitative polymerase chain reaction was used to measure the expression levels of miR-497 in 40 breast cancer specimens and adjacent normal breast tissues. MTT assays, colony formation assays, wound healing assays, transwell assays and cell cycle assays were used to explore the potential function of miR-497 in MDA-MB-231 breast cancer cells. Dual-luciferase reporter assays were performed to analyze the regulation of putative target of miR-497, and western blot assays were used to validate the dual-luciferase results.

**Results:**

The expression of miR-497 in breast cancer specimens was lower than adjacent normal tissues (*P* < 0.05). Overexpression of miR-497 inhibited cellular growth, suppressed cellular migration and invasion, and caused a G1 arrest. Dual-luciferase reporter assays showed that miR-497 binds the 3′-untranslated region (3′-UTR) of cyclin E1, suggesting that cyclin E1 is a direct target of miR-497. Western blot assays confirmed that overexpression of miR-497 reduced cyclin E1 protein levels.

**Conclusions:**

MiR-497 may act as a tumor suppressor gene in breast cancer. Inhibited cellular growth, suppressed cellular migration and invasion, and G1 cell cycle arrest were observed upon overexpression of miR-497 in cells, possibly by targeting cyclin E1. These results indicate miR-497 could be considered a therapeutic target for the development of treatment for breast cancer.

## Introduction

MicroRNAs (miRNAs) are approximately 22-nucleotide small RNAs that bind to the 3′-UTR of target mRNAs, causing gene silencing via translational repression or target degradation [1,2]. miRNAs are involved in many important physiological and pathological processes, such as cell proliferation, development, differentiation, virus infection and tumorigenesis, and are widely dysregulated in various cancers [1,3-5]. Hundreds of miRNAs have been discovered in mammals; however, the function of most miRNAs remains largely unknown. Previous studies showed that miRNA-497 (miR-497) was downregulated in several kinds of tumors, including human melanoma [6], gastric cancer [7] and adrenocortical carcinoma [8]. Similar results were also observed in breast cancer, in which the expression level of miR-497 was significantly lower in breast cancer patients and miR-497 levels inversely correlated with malignancy of breast cancer [9-11]. A recent study by Shen et al. suggested that miR-497 might induce apoptosis in MCF-7 breast cancer cells by targeting Bcl-w [9]. However, whether miR-497 regulates other target genes to control breast cancer cell growth and invasion is still unknown.

Cyclins are a family of proteins that control cell cycle progression by activating cyclin-dependent kinases (CDKs) and regulate the G1/S transition, DNA replication and mitosis. Cyclin E1 binds and activates CDK2 to promote the transition from G1 to S phase [12]. Previous studies showed that cyclin E1 overexpression was critical in the growth and survival of ovarian cancer cells and was significantly associated with poor prognosis [13]. Similarly, breast cancer patients with higher levels of cyclin E1 also showed higher mortality compared with low cyclin E1 patients [14-16].

In this study, we first demonstrated that the expression of miR-497 was significantly lower in breast cancer specimens compared with adjacent normal tissues. Overexpression of miR-497 inhibited cellular growth, suppressed cellular migration and invasion, and caused a G1 cell cycle arrest, likely by targeting cyclin E1. These results indicate that miR-497 may function as a tumor suppressor, and could be a therapeutic target for the development of future treatments for breast cancer.

## Results

### Expression of miR-497 was lower in breast cancer specimens than adjacent normal tissues

**Figure 1:**
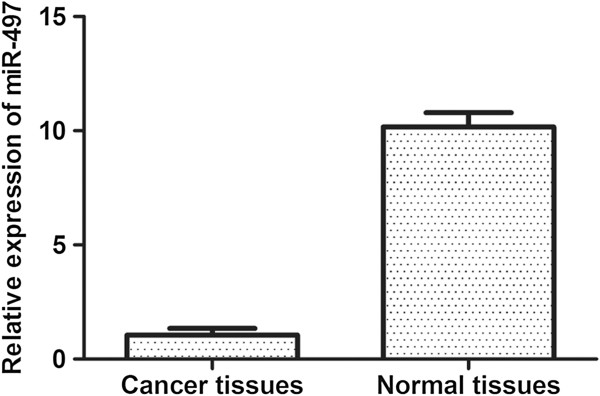
**MiR**-**497 levels are significantly decreased in breast cancer specimens.** The graph represents the 2^-ΔΔCt^ values ± SEM, *P < 0.05.

The expression levels of miR-497 were measured by quantitative polymerase chain reaction (qPCR) in forty breast cancer specimens and adjacent normal tissues. The results showed that the expression of miR-497 was lower in breast cancer specimens than adjacent normal tissues (Figure 1).

### Overexpression of miR-497 in MDA-MB-231 cells inhibited cell proliferation

**Figure 2:**
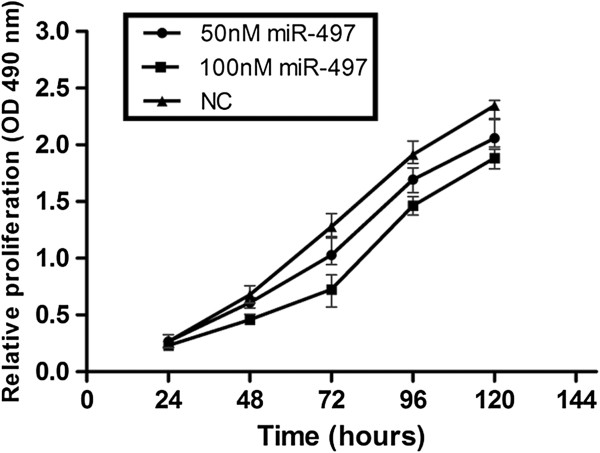
**MiR**-**497 expression inhibits MDA**-**MB**-**231 proliferation**. **MTT cell proliferation assays were performed with miR**-**497 and NC expressing cells.** The proliferation of MDA-MB-231 cells transfected with miR-497 was inhibited in a dose- and time-dependent manner compared with the NC group. The graph represents OD Values ± SEM, *P < 0.05.

**Figure 3:**
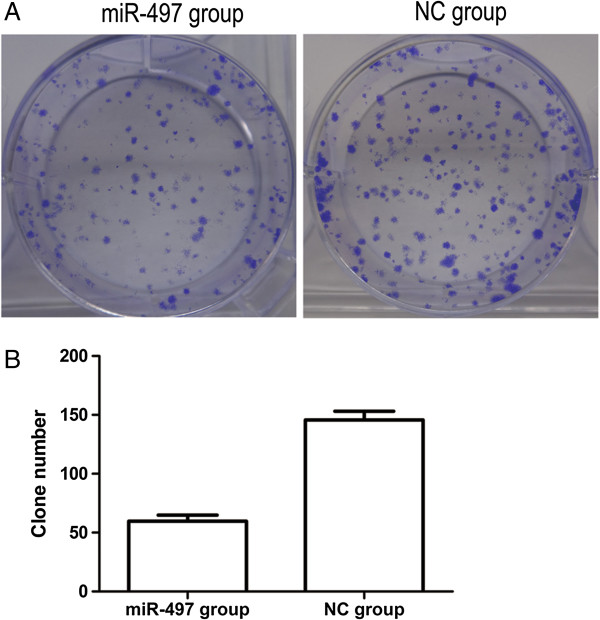
**Overexpression of miR**-**497 inhibited colony formation ability of MDA**-**MB**-**231 cells. (A)** Colony formation assays showed Crystal Violet staining of the miR-497-transfected group and NC-transfected group. **(B)** Graph indicates colony numbers of each experimental transfected group (60 ± 2 miR-497 group vs. 145 ± 2 NC group, *P < 0.05).

Next we examined the effects of miR-497 expression on cell proliferation by transfecting MDA-MB-231 cells with miR-497 or negative control (NC) mimics and performing MTT assays. Cells transfected with 50 nM and 100 nM miR-497 mimics groups showed significantly lower optical density (OD) values at 490 nm than the NC group from day 2 until day 5, in a time- and dose-dependent manner (Figure 2). Colony formation assays showed much less colony formation in the group transfected with 100 nM miR-497 compared with the NC group (Figure 3A, B). Together these results demonstrated that transient overexpression of miR-497 suppressed the colony formation ability of MDA-MB-231 cells, and indicate that overexpression of miR-497 suppressed cellular proliferation.

### Overexpression of miR-497 in MDA-MB-231 cells inhibited cell migration and invasion

**Figure 4:**
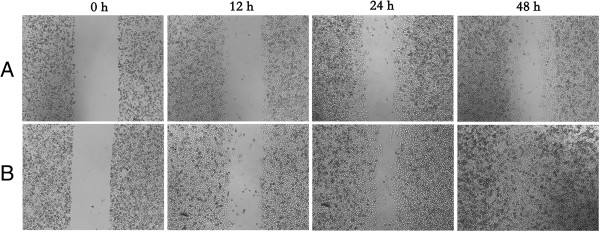
**Overexpressing of miR**-**497 in MDA**-**MB**-**231 cells showed impaired migration in wound healing assays. (A)** Image showed the gap of the scratched region of the miR-497-expressing MDA-MB-231 cells. **(B)** Image showed the region of the NC group cells.

**Figure 5:**
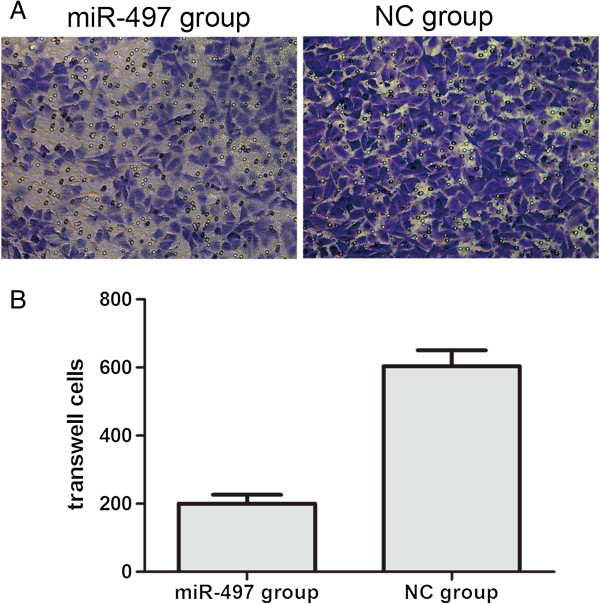
**MiR**-**497 overexpression affects invasion ability of MDA**-**MB**-**231 cells. (A)** Crystal Violet-stained invaded cells from transwell experiments. Images were obtained on an inverted microscope with × 200 magnification. **(B)** Invasion rates were determined by counting the number of cells invading through matrigel, *P < 0.05.

To study how overexpression of miR-497 affects cellular migration and invasion, we performed wound healing assays and transwell assays with MDA-MB-231 cells transfected with miR-497 mimics (100 nM) or NC mimics. The wound healing assay results showed that the migration ability of the miR-497 mimic groups was lower than the NC group. As shown in Figure 4, the cell-free area of the miR-497 group was significantly wider than the NC group at 24 h after drawing the “scratch” line on the monolayer cells. While the NC group filled in the gap at 48 h, the monolayer of miR-497-transfected cells still showed a clear gap in the scratched region. These results showed that overexpression of miR-497 in MDA-MB-231 cells inhibited cellular migration. In transwell invasion assays, the number of invaded cells stained with Crystal Violet was significantly less in the miR-497 (100 nM) group (Figure 5A). Invasion rates of both groups were determined by counting the number of cells that invaded through matrigel and confirmed the results observed by inverted microscope (Figure 5B). Together these results indicated that overexpression of miR-497 can suppress cellular migration and invasion ability.

### Cell cycle was affected following overexpression of miR-497 in MDA-MB-231 cells

**Figure 6:**
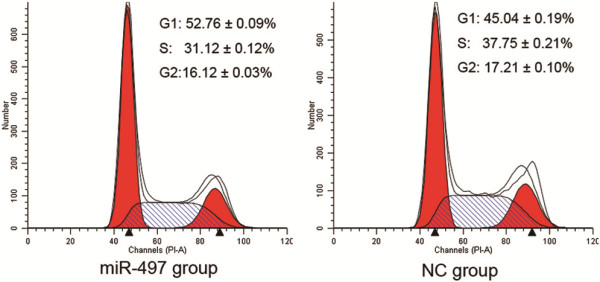
**Overexpression of miR**-**497 in MDA**-**MB**-**231 cells caused G1 phase accumulation.** MDA-MB-231 cells transfected with miR-497 or NC mimics were analyzed by flow cytometry. The respective proportion of G1 phase, S phase and G2 phase cells of miR-497 and NC groups are indicated.

The cell cycle distributions of the miR-497 (100 nM) group and NC group were analyzed by flow cytometry. As shown in Figure 6, the percentage of miR- 497-transfected cells remaining in G1 phase was significantly higher than the NC group (52.76 ± 0.09% vs. 45.04 ± 0.19%, respectively, *P* < 0.05), with approximately 7.72% more cells in G1 phase in the miR-497 group compared with the NC group. These results indicated that the overexpression of miR-497 could impact cell cycle progression in MDA-MB-231 cells.

### MiR-497 targets cyclin E1 and regulates its expression in MDA-MB-231 cells

**Figure 7:**
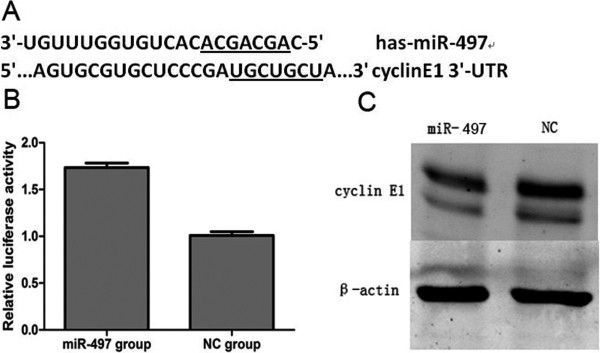
**miR-497 targeted cyclin E1. (A)** The miR-497 binding site in cyclin E1 3′-UTR, located 247–254 bp upstream of the cyclin E1 3′-UTR. **(B)** The relative luciferase of activity in miR-497 group and NC group (*P < 0.05). **(C)** Western blot analysis of cyclin E1 protein levels in miR-497-overexpressing and NC cells.

We searched for potential targets of miR-497 using several online databases, including targetscan, miRanda, miRBase and miRGen, and all four databases indicated that the cyclin E1 mRNA contained miR-497 binding sites. To examine the possibility that miR-497 targets cyclin E1, we used dual-luciferase reporter assays, in which the reporter activity is evaluated by normalizing the Renilla luciferase (RL) activity with firefly luciferase (FL) activity, the FL/RL ratio indicates the relative activity levels. We constructed a psiCHECK-2/cyclin E1 3′-UTR vector, which contains the RL gene and the 3′-UTR region of cyclin E1. Cells were transfected with either miR-497 or NC mimic, along with psiCHECK-2/cyclin E1 3′-UTR, and luciferase activity was analyzed. The FL/RL ratio in the miR-497 group was approximately 1.5-fold higher than the NC group (*P* < 0.05) (Figure 7B). Results were consistent over three independent experiments. These results show that miR-497 could directly interact with the cyclin E1 3′-UTR in the psiCHECK-2 reporter plasmid, leading to the degradation of RL mRNA.

Furthermore, western blot analysis (Figure 7C) showed that cyclin E1 protein levels were lower in the miR-497-overexpressing group compared with the NC group. Taken together, these results indicate that miR-497 could directly bind to the cyclin E1 3′-UTR region to regulate cyclin E1 in MDA-MB-231 cells. Figure 7A shows the binding site of miR-497 to cyclin E1 3′-UTR is located 247–254 bp upstream of the cyclin E1 3′-UTR.

## Discussion

The study on the roles of miRNA in the development of tumors has become a subject of intense investigation. It is estimated that approximately 30% of human genes are miRNA targets [17]. One miRNA may have multiple different mRNA targets, and at the same time, one mRNA might be targeted by multiple miRNAs. If the miRNA is perfectly complementary to its target, it can specifically cleave the target mRNA. However, if it is not perfectly complementary to its target, the miRNA will inhibit mRNA translation. Thus, the nature of the interactions between miRNAs and their target genes forms a complicated regulatory network [18,19]. Most miRNAs involved in the regulation of tumor growth function through the two pathways discussed above.

In this study, we examined the expression levels of miR-497 in breast cancer specimens and adjacent normal tissues. We found that the expression of miR-497 was significantly lower in breast cancer specimens, which suggested that the expression of miR-497 was associated with the development of breast cancer. Thus, we hypothesized that miR-497 may function as a tumor suppressor. Shen et al. reported that upregulation of miR-497 expression in MCF-7 breast cancer cells causes cellular growth inhibition, apoptotic enhancement and G0/G1 phase arrest [9], in our study, we transfected miR-497 mimics into MDA-MB-231 cells to induce its overexpression. Exogenous overexpression of miR-497 significantly inhibited the cell growth as indicated by MTT assays and colony formation assays. Moreover, cell migration and invasion abilities were also significantly reduced by overexpression of miR-497 in MDA-MB-231 cells, as shown by wound healing assays and transwell assays. Furthermore, analysis of miR-497 mimic cells by flow cytometry showed that miR-497 induced G1 arrest compared with NC cells.

Our results have shown that overexpression of miR-497 in breast cells greatly influences cell proliferation, migration, invasion and cell cycle progression. We searched for potential targets of miR-497 using several online databases, including targetscan, miRanda, miRBase and miRGen, and all four databases indicated that the cyclin E1 mRNA contained miR-497 binding sites. The interaction between miR-497 and cyclin E1 mRNA has not been previously reported. To test whether cyclin E1 is a real target of miR-497, we constructed the psiCHECK-2/ cyclinE1 3′-UTR plasmid, which contains the 3′-UTR of cyclin E1. Through dual-luciferase assays, we confirmed the cyclin E1 3′-UTR as a direct target of miR-497 in MDA-MB-231 cells. Additionally, we found that the protein levels of cyclin E1 were significantly lower in miR-497-overexpressing cells than those in NC cells. Together, these findings support the prediction that cyclin E1 is a downstream target of miR-497.

In summary, our findings suggest that miR-497 can specifically regulate cell growth and invasion by targeting cyclin E1 in MDA-MB-231 cells. Its overexpression can inhibit cellular migratory ability and disrupt cell cycle progression. Thus, these results indicate that miR-497 may acts as a tumor suppressor in breast cancer. Moreover, the dual-luciferase and western blot assays identify cyclin E1 as a downstream target of miR-497. The artificial upregulation of miR-497 using cyclin E1 as a therapeutic agent could offer a promising new direction for future breast cancer treatment.

## Materials and methods

### Specimens

Forty paired breast cancer specimens and adjacent normal breast tissues were collected from the Department of General Surgery of the Shanghai Tenth People’s Hospital. The samples were immediately snap-frozen in liquid nitrogen. All samples were confirmed as invasive, ductal breast cancer by trained pathologists. No patients received chemotherapy or radiotherapy prior to surgery. The collection of the patient specimens were approved by Institutional Ethics Committees of Tongji University, the approval number: SHSY - IEC - pap3.0/13-3.

### Cell culture and transfection

Human MDA-MB-231 breast cancer cells and human embryonic kidney 293T cells were obtained from the Chinese Science Institute. The cells were cultured in Dulbecco’s modified Eagle’s medium (DMEM; Gibco, USA) supplemented with 10% fetal bovine serum (FBS; Gibco), penicillin (100 units/ml) and streptomycin (100 μg/ml) (Enpromise, Hangzhou, China). Cells were incubated at 37°C in a humidified chamber supplemented with 5% CO_2_. Cells at approximately 90% confluence were split at a 1:2 ratio every 3 days.

Cells (2 × 10^5^) were added into each well of a 6-well plate and cultured with DMEM medium without either serum or antibiotics. When MDA-MB-231 cell density reached 30–40%, cells were transfected using lipofectamine transfection reagents (Invitrogen, USA), according to the manufacturer’s instructions. MiR-497 and NC mimics were purchased from Gene Pharma (Shanghai, China), and sequences are as follows: miR-497, 5′-CAGCAGCACACUGUGGUUUGU-3′, and NC, 5′-UUGUACUACACAAAAGUACUG-3′. After 5–6 h of incubation, DMEM medium was replaced by DMEM with 10% FBS.

### MicroRNA isolation and quantitative polymerase chain reaction (qPCR)

miRNAs of forty paired breast cancer specimens and adjacent normal breast tissues were extracted using the miRcute microRNA isolation kit (Tiangen, Beijing, China), according to the manufacturer’s instructions. Expression levels of miR-497 were analyzed by using one-step qRT-PCR (EzOmics SYBR qPCR kit); the miR-497 stem-loop primer, U6 primer and EzOmics SYBR qPCR kit were all purchased from Biomics Biotechnology Inc (Jiangsu, China). Real-time PCR was performed on a 7900HT fast real time-PCR instrument (Applied Biosystems, Singapore) using the following primers: miR-497:

5′-GTCGTATCCAGTGCAGGGTCCGAGGTATTCGCACTGGATACGACACAAA-3′ (stem-loop primer), 5′-CGCCAGCAGCACACTGTGG-3′ (sense) and 5′-GTGCAGGGTCCGAGGT-3′ (antisense); U6:

5′-GTCCTATCCAGTGCAGGGTCCGAGGTGCACTGGATACGACAAAATATGGAAC-3′ (stem-loop primer), 5′-TGCGGGTGCTCGCTTCGCAGC-3′ (sense) and 5′-CCAGTGCAGGGTCCGAGGT-3′ (antisense). RNA (100 ng) was added in a 25 μl reaction mixture containing 12.5 μl 2× Master mix, 0.5 μl 50× SYBR Green, 0.5 μl reverse transcription primer (10 μM), and 0.5 μl sense and 0.5 μl antisense primers (10 μM) for miR-497. One step PCR parameters for miRNA quantification were as follows: 37°C for 60 min for reverse transcription, 10 min at 95°C, followed by 40 cycles of 20 sec at 95°C, 30 sec at 62°C and 30 sec at 72°C. Ct values were collected at the end of the PCR. Each sample was tested in triplicate, and the relative quantification equation was used to calculate the relative expression.

### Cell proliferation assay (MTT assay)

Cells (2 × 10^3^) were plated in 96-well plates (BD Biosciences, USA) and incubated at 37°C until the cells reached 30–40% confluence, followed by transfection with 50 nM or 100 nM miR-497 or NC mimics. Cell proliferation was assessed at 24, 48, 72, 96 and 120 h as follows: 20 μl (5 mg/ml) MTT solution (Sigma, USA) was added in each well, and after 4 h of incubation at 37°C, the supernatant was discarded and 150 μl of dimethyl sulfoxide (DMSO) were added. After 10 min of low speed shaking (100 rpm) and incubation, the OD at 490 nm was read by a microplate spectrophotometer. Each sample was tested with six replicates. All experiments were performed in biological triplicate.

### Colony formation assay

After transfection with 100 nM miR-497 or NC mimics, 400 cells were plated in a 6-well plate in complete medium, and the plate was shaken to disperse the cells equally. After incubation at 37°C with 5% CO_2_ for 7–10 days, when the colonies were visible by eye, the culture was terminated by removing the medium and washing cells twice with phosphate-buffered saline (PBS). The colonies were fixed with 95% ethanol for 10 min, dried and stained with 0.1% Crystal Violet solution for 10 min, and the plate was washed three times with water. Images were taken of the stained plates, and the numbers of colonies containing more than 50 cells were counted. Each treatment was performed in triplicate.

### Cell migration and invasion assay

Cell migration was evaluated by the wound healing assay, also known as the “scratch” assay. MDA-MB-231 cells were transfected with miR-497 mimics (100 nM) or NC mimics, and when cells reached 90% confluence, a scratch was made through each well using a sterile pipette tip. Cells were monitored under the microscope (magnification, ×50) for 0, 12, 24 and 48 h after wounding. Images of cells were captured at the same position before and after incubation to document the repair process. The experiments were repeated three times.

Transwell invasion assays were performed to evaluate invasion ability as follows: 1 ml 10% FBS DMEM was added into a 24-well plate, and a transwell filter insert was placed into the well; the filter was filled with 5 × 10^4^ cells (miR-497-transfected or NC-transfected cells) in 200 μl DMEM with 0.1% BSA, and the cells were cultured for 30 h at 37°C in 5% CO_2_. Invasion was observed under an inverted microscope, and when the cells crossed into the basal well, the invasion was terminated. The matrigel was scraped off, and numbers of cells remaining in the basal well were analyzed. The filters were washed three times with PBS, fixed with 4% paraformaldehyde, stained by 0.1% Crystal Violet solution, and washed three times with water. Images of the stained cells were obtained. To quantify the number of invading cells, Crystal Violet-stained cells in 10 random visual fields were counted, and means were obtained for statistical analysis.

### Cell cycle analysis

MDA-MB-231 cells transfected with miR-497 (100 nM) or NC mimics were harvested 36 h after transfection, centrifuged at 1,200 rpm for 10 min and washed three times with cold PBS. Ice-cold 70% ethanol was added dropwise, and cells were fixed in 4°C overnight. After 30 min digestion by RNase (0.1 g/L), 250 μl propidium iodide staining solution (0.05 g/l) was added to each sample. Cells were incubated for 30 min at room temperature in the dark and analyzed by a flow cytometer (FACSCanto™ II, BD Biosciences).

### Dual-luciferase reporter assay

293T cells were seeded in 12-well plates (BD, USA) and cultured until the cells reached 80–90% confluence. The Cyclin E1 3′-UTR was cloned into the psiCHECK-2 vector, which contains the RL gene, and co-transfected into cells together with miR-497 or NC mimics (100 nM) using lipofectamine, according to the manufacturer’s instructions. Thirty hours after transfection, luciferase activity was measured using the Dual-Luciferase Reporter assay kit (Promega, USA). Briefly, the cells were washed twice with PBS, lysed with passive lysis buffer and incubated at room temperature for 15 min. The supernatants were collected and 20 μl were added into 96-well plates. The FL reporter was measured immediately after adding luciferase assay reagent II (LAR II). After quantifying the firefly luminescence, 100 μl Stop&Glo® Reagent was added to each well to initiate the RL reporter and Renilla luminescence was then measured. The data were analyzed by normalizing RL with FL, and the ratio of FL/RL was calculated to indicate the activity of the reporter.

### Western blot analysis

Cells were washed twice by cold PBS, and RIPA lysis buffer was added (1% Triton X-100, 50 mmol/L Tris pH 7.4, 150 mmol/L NaCl, 20 mmol/L Iodoacetamide, 1 mmol/L Phenylmethanesulfonyl fluoride and 1% aprotinin). Cells were lysed on ice for 30 min, the cell lysate was collected into microtubes, and samples were centrifuged for 15 min at 12000 rpm at 4°C. Supernatants were collected and the protein concentrations were measured using the BCA Protein Assay Kit (Beyotime, Jiangsu, China). Protein samples (25 μg) were denatured with 5× sodium dodecyl sulfate (SDS) loading buffer (100 mmol/L Tri-HCl pH 6.8, 4% SDS, 0.2% bromophenol blue, 20% glycerin, 200 mmol/L β-mercaptoethanol) at 95°C for 5 min. Protein samples were separated on a 10% SDS polyacrylamide gel electrophoresis and transferred onto 0.45 μm nitrocellulose membranes (Beyotime). Following 60 min of blocking with 5% fat-free milk, membranes were incubated with cyclin E1 antibody (1:1000, Epitomics, USA) and β-actin antibody (1:1000, Epitomics) overnight at 4°C. Blots were washed and incubated for 1 h with the anti-rabbit secondary antibody (1:1000, Epitomics). After three washes with PBST, immunoreactive protein bands were detected with an Odyssey Scanning system (Li-Cor, Lincoln, NE, USA).

### Statistical analysis

Data are presented as the mean ± standard error of mean from at least three independent experiments. The two-tailed *t*-test was used to draw a comparison between groups. The null hypothesis was rejected at the 0.05 level.

## Abbreviations

miRNA: microRNA; NC: Negative control; qPCR: quantitative polymerase chain reaction; nM: nmol/l; FBS: Fetal bovine serum; DMEM: Dulbecco’s modified Eagle’s medium; RL: Renilla luciferase; FL: Firefly luciferase.

## Competing interests

The authors declare that they have no competing interests.

## Authors’ contributions

LF designed and directed the study. XL performed cell proliferation and colony formation assays. YL, QL and YG performed qRT-PCR and cell cycle assays. LC and YH conducted western blotting assays. QL performed dual-luciferase reporter assays, cell migration and invasion assays, and drafted the manuscript. All authors read and approved the final manuscript.
